# Socio-cultural factors explaining timely and appropriate use of health facilities for *degedege *in south-eastern Tanzania

**DOI:** 10.1186/1475-2875-8-144

**Published:** 2009-06-29

**Authors:** Angel Dillip, Manuel W Hetzel, Dominic Gosoniu, Flora Kessy, Christian Lengeler, Iddy Mayumana, Christopher Mshana, Hassan Mshinda, Alexander Schulze, Ahmed Makemba, Constanze Pfeiffer, Mitchell G Weiss, Brigit Obrist

**Affiliations:** 1 Ifakara Health Institute, Off Mlabani Passage PO Box 53, Ifakara, Morogoro, Tanzania; 2 Papua New Guinea Institute of Medical Research, Goroka, EHP 441, Papua New Guinea; 3 University of Basel, Swiss Tropical Institute, Socinstrasse 57, CH-4002, Basel, Switzerland; 4 Novartis Foundation for Sustainable Development, WRO-1002.11.56, CH-4002 Basel, Switzerland; 5 Tanzania Commission for Science and Technology, PO Box 4302, Dar es Salaam, Tanzania

## Abstract

**Background:**

Convulsions is one of the key signs of severe malaria among children under five years of age, potentially leading to serious complications or death. Several studies of care-seeking behaviour have revealed that local illness concepts linked to convulsions (referred to as *degedege *in Tanzanian Kiswahili) called for traditional treatment practices while modern treatment was preferred for common fevers. However, recent studies found that even children with convulsions were first brought to health facilities. This study integrated ethnographic and public health approaches in order to investigate this seemingly contradictory evidence. Carefully drawn random samples were used to maximize the representativity of the results.

**Methods:**

The study used a cultural epidemiology approach and applied a locally adapted version of the Explanatory Model Interview Catalogue (EMIC), which ensures a comprehensive investigation of disease perception and treatment patterns. The tool was applied in three studies; i) the 2004 random sample cross-sectional community fever survey (N = 80), ii) the 2004–2006 longitudinal *degedege *study (N = 129), and iii) the 2005 cohort study on fever during the main farming season (N = 29).

**Results:**

71.1% of all convulsion cases were brought to a health facility in time, i.e. within 24 hours after onset of first symptoms. This compares very favourably with a figure of 45.6% for mild fever cases in children. The patterns of distress associated with less timely health facility use and receipt of anti-malarials among children with *degedege *were generalized symptoms, rather than the typical symptoms of convulsions. Traditional and moral causes were associated with less timely health facility use and receipt of anti-malarials. However, the high rate of appropriate action indicates that these ideas were not so influential any more as in the past. Reasons given by caretakers who administered anti-malarials to children without attending a health facility were either that facilities were out of stock, that they lacked money to pay for treatment, or that facilities did not provide diagnosis.

**Conclusion:**

The findings from this sample from a highly malaria-endemic area give support to the more recent studies showing that children with convulsions are more likely to use health facilities than traditional practices. This study has identified health system and livelihood factors, rather than local understandings of symptoms and causes relating to *degedege *as limiting health-seeking behaviours. Improvements on the supply side and the demand side are necessary to ensure people's timely and appropriate treatment: Quality of care at health facilities needs to be improved by making diagnosis and provider compliance with treatment guidelines more accurate and therapies including drugs more available and affordable to communities. Treatment seeking needs to be facilitated by strengthening livelihoods including economic capabilities.

## Background

Malaria continues to be a major public health problem worldwide and one of the leading causes of morbidity and mortality in sub-Saharan Africa, despite global and national control efforts. Malaria is one of the five life-threatening conditions causing more than half a million deaths of African children annually [1]. In Tanzania, malaria is responsible for 34% of deaths among children under five years of age [2]. The global strategy for malaria control mainly focuses on case management, through early diagnosis and prompt treatment of all fever cases in malaria endemic areas [1]. Prompt diagnosis and timely malaria treatment within 24 hours after onset of first symptoms can reduce illness progression to severe stages, and therefore, decrease mortality.

Recent studies point out that before referral rectal artesunate can be given to patients who cannot be treated in time and are several hours away from facilities [3]. If patients cannot access injections or be treated orally, a single artesunate suppository at the time of the referral can reduce the risk of death or permanent disability. Nevertheless, the same multi-country trial showed that there was only a reduction of mortality of patients that were treated no later than six hours. Reaching clinics in time still remains crucial. However, prompt malaria treatment is a major challenge in many developing countries [4].

Before developing a partial immunity, children below five years of age in endemic countries are at a high risk of acquiring a severe form of malaria. Besides contributing to death, severe malaria amongst children under five years of age may cause serious complications, including brain damage and anaemia. The standard treatment of severe malaria is an intramuscular injection or an intravenous infusion of quinine [5].

Convulsions are one of the main manifestations of severe malaria. Several studies on childhood illnesses as reviewed by Williams and Jones [6] have provided contradictory evidence with regard to treatment of convulsions. Research in sub-Saharan Africa has revealed that conditions with convulsions as leading symptoms were not linked to malaria, and were primarily treated with traditional practices, as opposed to common fever cases for which modern biomedical treatment was preferred [7-12].

In Tanzania, the Kiswahili term used for convulsions in children is *degedege*. '*Degedege*', meaning '*bird bird'*, is believed to be an evil spirit that takes form of a bird and casts its shadows on children who eventually become ill, develop convulsions and may die [13-15]. Fever cases are referred to as *homa*, and are commonly associated with malaria. Although the link between malaria and convulsions is recognized, *degedege *and *homa *tend to be treated differently. *Degedege *is reported more likely to be managed by traditional practices, while *homa *is mainly treated by the formal health system [14,16-19]. On the other hand, a study conducted in Kilosa and Handeni Districts in Tanzania [20] found that traditional healers encouraged modern treatment-seeking for convulsing children by referring severe cases of malaria to health facility. A recent study using verbal autopsy methodology indicated that 78.7% of malaria deaths in south-eastern Tanzania used modern treatment as the first resort to care [21]. Similarly, vignette interviews in Ghana revealed that more than 90% of respondents identified convulsions as severe conditions and recognized the need to seek modern treatment [22].

Some studies on the other hand showed that children with *homa *and/or *degedege *did not attend health facilities due to a number of access related factors, including availability and affordability of services [23-25]. Health service studies focus on health system factors and reducing supply barriers to ensure access to health care [26-28]. Such studies concentrate on improving the quality of care through ensuring availability of supplies, skilled staff, and quality of service [29].

The present study compared the local understanding and treatment of children under five years of age suffering from *degedege *with fever cases in rural areas of south-eastern Tanzania. Children under five years were selected because they are most at risk of contracting malaria. Within the study, interviews with caretakers (parents/guardians) were conducted. The objective of the study was to describe and explain first treatment-seeking for *degedege*, and to explore how understanding of symptoms and causes related to *degedege *influence response strategies of care takers.

This study was conducted within the framework of the ACCESS Programme, a malaria intervention aiming at understanding and improving access to prompt and effective malaria treatment in rural Tanzania [4]. The study complements previous research [30] carried out within the same programme to investigate access obstacles to malaria treatment.

## Methods

### Study area

This study took place in the Kilombero and Ulanga Districts in Southeastern Tanzania. The research site is part of the local Health Demographic Surveillance System (HDSS) covering 25 villages in the two districts. The programme conducts its monitoring and evaluation activities through this HDSS. Every four months HDSS staff monitor and register all births, deaths, pregnancies, socio-economic indicators, and in- and out-migrations of all residents through household surveys [4]. This allows on one hand the tracking of all individuals and most importantly for this work, to draw an almost perfect random sample, which is essential for the representativity of the results. In 2004, the population of the 25 HDSS villages in Kilombero and Ulanga Districts was 74,200 inhabitants, with an additional 45,700 people living in Ifakara town, the main settlement in the districts [31].

In 2004 there were 14 public and private health facilities in the study area [4]. Moreover over 400 drug shops were present (general shops selling drugs: 454; shops stocking anti-malarials: 54); from 2006 onwards these included among which Accredited Drug Dispensing Outlets (ADDOs), which are allowed to sell a limited range of prescription-only medicine, and so-called Part II drug stores, which offer over-the-counter (OTC) drugs. General shops provide antipyretics, although in some villages they illegally dispense anti-malarials as well. There are many traditional healers providing varying kinds of services ranging from protection of disease, to treatment of illness and other misfortunes associated with witchcraft and spirits [13].

The most common activities are subsistence farming, fishing and small scale trading, with rice and maize being the predominant food crops. Families spend several months in their farming site (*shamba *in Kiswahili), located at an average 7.5 km away from the main houses in the fertile lower wetlands [32].

### Instruments

The present study used a cultural epidemiology approach, and applied a locally adapted and constructed version of the Explanatory Model Interview Catalogue [33]. EMIC interviews allow for assessing specified health problems from the local perspective of affected individuals, their families, and/or community members [22]. The interviews aim to gather information on people's knowledge, understanding and experience of diseases such as malaria [13,16]. As a semi-structured interview, the EMIC was applied in the ACCESS Programme in order to collect both quantitative and qualitative information on illness experience, its meaning and related behaviour for fever and *degedege*, mostly referred to as patterns of distress (PD), perceived causes (PC) and help-seeking (HS) [33]. EMIC interviews extract explanatory models of respondents in their own terms [33] and explain how such models influence particular behaviors. For the purpose of this study, EMIC was employed to understand how explanatory models for *degedege *influence treatment-seeking behaviours.

### Study design

A similar EMIC was applied in three different studies (for details see Table 1):

**Table 1:** Characteristics of the Three Studies

	Cross-sectional Community fever survey	Longitudinal *degedege *community study	Longitudinal *shamba *(farm sites) fever study
**Aim of the study**	To examine treatment-seeking behaviour for fever	To explain treatment-seeking behaviour for *degedege*, and identify how symptoms and causes related to *degedege *effect first response strategies	To examine treatment-seeking for fever during farming season
			

**Study setting**	HDSS area of Kilombero and Ulanga	HDSS area of Kilombero and Ulanga	HDSS area of Kilombero and Ulanga
			

**Sample size**	80	129	29

**Sampling procedure**	A total number of 318 households were random-sampled from the registered 16,220 households in the DSS villages. Only households with at least one under five years of age child were included in the study. Of all 318 households, 58 under five years of age households reported a fever episode in the past 14 days. In Ifakara town, a two stage random sampling of 223 households was performed. Of all 329 ten-cell leaders, a random sample of 35 ten cell leaders were visited to obtain a complete list of their ten cells. Six households per each ten-cell were randomly sampled. Only 22 households with children under five years of age met the selection criteria.	129 *Degedege *cases were continuously extracted from DSS records between November 2004 to March 2006 and followed up for an EMIC interview	Ten out of 25 HDSS villages were randomly selected. A two stage random sample of 159 households was drawn in proportion to the relative size of the village. Altogether 100 households were eligible, but only 29 households included children under five years of age who had recovered from a recent fever episode. These households were therefore followed for an EMIC interview on monthly basis

**Study respondents**	Caretakers of children under five years of age	Caretakers of children under five years of age	Caretakers of children under five years of age

**Interviewers**	ACCESS and HDSS Staff	ACCESS Staff	ACCESS Staff

1. A random sample cross-sectional community fever survey drawn from the HDSS and Ifakara Town areas (N = 80)

2. The longitudinal *degedege *community study with cases drawn from the longitudinal HDSS surveillance (N = 129)

3. Longitudinal *shamba *fever study in which a random sample of households was followed during the time spent in their *shamba *(N = 29).

All studies only included children who had experienced either an episode of fever or of *degedege *in the past 14 days. For ethical reasons, children who had not recovered the day before the interview were excluded from the study, and those who were still sick at the time of the interviewer's visit were advised to attend a health facility. The EMIC was administered to caretakers of the children immediately after finding the case, except for the longitudinal *degedege *study, in which interviewers visited the household of the child two to four weeks after a HDSS field worker had identified and reported the illness episode. A caretaker was defined as the parent/guardian who provides the daily essential needs of the child, such as bathing, feeding, clothing, or sending the child to school or to the hospital when sick.

Interviews were conducted in Kiswahili by HDSS field workers and by two trained local interviewers of the ACCESS programme, one doing the interview, a second one recording the answers. All interviews, except those done by the HDSS field workers, were tape-recorded, transcribed and entered into their respective sections in the EMIC. The HDSS interviews were entered directly into the interview questionnaire (EMIC) without being recorded.

For the purpose of adhering to these ethical requirements, all participating individuals who were involved in this project were informed about its aim, the interview procedures, and about their rights as informants before they were asked for their verbal consent. Participation in data collection was voluntary and participants were free to leave at any time.

### Sample

(1) For the HDSS community fever survey, a village stratified random sample was drawn whereby the number of households sampled was proportional to the total number of households in the village. In total, 318 households were drawn from the registered 16,220 households in the DSS villages [30]. Only households with at least one child under five years of age were included in the study. Of all 318 households, 58 reported a fever episode in the past 14 days for a child under five years of age. These households were visited between May 2004 and August 2004 for an EMIC interview.

In Ifakara town, there was no updated household list available for sampling purposes. With the support of village and hamlet leaders, the majority of households could be identified and a two-stage random sampling of 223 households performed. Since every household is supposed to be under a ten cell leader a list of all 329 ten cell leaders was established with support from local government officials [30]. A random sample of 35 ten cell leaders was visited for the purpose of setting up a complete list of households within their ten-cells. Six households from each ten cell were then randomly sampled for the study. Only 22 households with children under five years of age met the selection criteria and were visited between May 2004 and August 2004 for an EMIC Interview.

(2) The longitudinal *degedege *study was conducted between November 2004 and March 2006 in the area of the HDSS. A total number of 129 households whose children had *degedege *were extracted continuously from DSS records and followed up for an EMIC interview.

(3) For the *shamba *study, data were collected between January and August 2005, to examine treatment-seeking patterns during the farming season. Ten out of 25 HDSS villages were randomly chosen for the study. A list of villagers (5,912) who had reported to own a farming plot was obtained from HDSS records. A two stage random sample of 159 households was drawn in proportion to the relative size of the village. Altogether 100 households were eligible for the study, but only 29 households had children below five years of age who had recovered from a fever episode. These households (29) were identified and followed to their farming fields on a monthly basis for an EMIC interview.

### Data management and analysis

Quantitative data from the EMIC interviews were double-entered using Microsoft Fox Pro, and analysed using SAS software (Statistical Analysis of Software Institute, Cary, NC, USA). The analysis was re-computed with reference to illness labels: *homa*, malaria and *degedege*. Referring to the national malaria treatment policy, which recommends treatment of malaria within 24 hours of onset of symptoms, children were classified with respect to three outcome variables: (1) 'Timely health facility use' (HF use), defined to include health facility use within 24 hours; (2) 'Timely health facility and anti-malarial use' (HF AM) meaning health facility and anti-malarial use within 24 hours; (3) 'Timely anti-malarial not from the health facility' (AM not HF) refers to children who used timely anti-malarial not received from a health facility. The correlation of socio-demographic variables, patterns of distress (illness experiences), and perceived causes (meaning of illness) with the outcome variables were analysed and adjusted in a multivariate logistic regression model.

To clarify the nature of the explanatory model variables and how these explain the outcome variables, the MaxQDA software was used [34]. A coded template for each study was prepared in Microsoft Word, through which qualitative narratives from EMIC interviews were entered before being imported into MaxQDA. Relevant variables were imported from the quantitative data set to select records of particular interest. Content analysis of illness narratives detailed the meaning of categorical codes, and explained the character of correlation identified from quantitative analysis.

## Results

### Sample characteristics

The socio-demographic characteristics of respondents from the three studies are presented in Table 2. The analysis included 235 children whose caretakers identified the condition as malaria, *homa *or *degedege*; three cases that did not fall into any of the above illness labels were excluded. The majority of family caretakers interviewed were women (82.6%) and married (80%). 41.3% of respondents disclosed that their income is not regular or dependable. The majority (94.9%) depended on farming as the main source of income and livelihood. Regarding the place of illness recognition, 73.6% of caretakers recognized the illness of the children at home.

**Table 2:** Demographic Characteristics of Caretaker Respondents (%)

	*Degedege *n = 135	Malaria n = 72	*Homa *n = 28	Total n = 235
**Relationship to the child**				
Mother	85.9	77.8	78.6	82.6
Father	10.4	15.3	17.9	12.8
Grandmother	0.7	4.2	0.0	1.7
Other	3.0	1.4	0.0	2.1
Not specified	0.0	1.3	3.5	0.8
**Marital status**				
Never married	9.6	13.9	10.7	11.1
Married	83.0	77.8	71.4	80.0
Separated, divorced	5.9	8.3	14.3	7.7
Widowed	0.7	0.0	0.0	0.4
Not specified	0.8	0.0	3.6	0.8
**Income**				
Regular and dependable	40.0	30.6	42.9	37.4
Possibly	17.0	8.3	7.1	13.2
Uncertain	10.4	6.9	0.0	8.1
Not regular and dependable	32.6	54.2	50.0	41.3
**Occupation**				
Farmer	94.8	94.4	96.4	94.9
Trade/Business	3.7	4.2	3.6	3.8
Labourers	1.4	0.0	0.0	0.8
Teacher	0.0	1.4	0.0	0.4
**Illness recognition**				
Home	79.3	72.2	50.0	73.6
*Shamba*	20.7	27.8	50.0	26.4

With regard to treatment options for the children, 60.9% of all children with *homa*, malaria or *degedege *were reported to have 'timely health facility' use (HF use). Out of these, 57% obtained anti-malarial medicines from the facility (HF AM), and 23% used 'timely anti-malarials not from health facility' (AM not HF) (Table 3).

**Table 3:** Demographic and socio-cultural features of children with *degedege*, malaria and *homa*(n = 235)

	HF Use ^1)^ n = 143 (60.9%)		HF AM ^2)^ n = 134 (57%)		AM not HF ^3)^ n = 54 (23%)	
	Estimate	P-Value	Estimate	P-Value	Estimate	P-Value
**Demographics**						
Degedege*	0.69	**0.00**	0.41	0.06	-0.39	0.19
HF availability					0.61	**0.04**
HF immediate use (only for AM not HF)					-1.69	**0.00**
Female Sex	-0.78	**0.02**	-0.82	**0.02**		
Never Married					-1.98	**0.02**
Sep/Div					-1.14	0.21
Farmer	-2.89	**0.02**	-1.91	**0.04**	-1.57	0.10
**Categories of distress**						
No interest to play	0.88	**0.01**	0.89	**0.01**		
Not happy					1.41	**0.03**
Sleeps					-1.29	**0.00**
Loss of appetite			-0.24	0.16	-0.42	0.09
Crying all the time	-0.37	0.15				
Hot body					0.28	0.07
Hot abdomen	0.44	**0.04**	0.39	0.07	-1.68	**0.00**
Cough	0.65	**0.02**	0.69	**0.01**		
Difficult breathing	0.53	**0.02**	0.60	**0.01**		
Yellow eyes					0.71	0.08
Diarrhoea	0.29	0.07			0.49	**0.00**
Shivering					0.59	**0.05**
Body becomes stiff	0.39	0.05	0.33	0.09		
Froth in the mouth					-1.15	**0.05**
Easily startled	-0.34	0.27				
**Perceived causes**						
Impure water			0.38	0.07		
Eating leftover food	1.19	**0.00**	1.51	**0.00**		
Breast feeding					2.59	**0.00**
Worms	-0.56	0.13	-0.50	0.19	0.92	0.08
Stage of child growth	0.76	**0.01**	0.83	**0.01**	0.60	0.09
Constitution/blood weakness	0.77	0.13	1.14	**0.03**		
Hereditary	-1.87	**0.01**	-1.85	**0.01**	-1.99	0.05
Sanitation/dirty environment					-0.45	0.08
Contamination – contact			-1.09	**0.03**		
Wind					1.02	0.11
Failure to abstain from sex (parent)	-0.91	0.13	-0.75	0.16	-1.41	0.06
Bird/insect called *degedege*	-0.31	**0.05**	-0.37	**0.02**	-0.80	**0.04**
Other mention			0.16	0.16	-0.17	0.43
Don't know					0.94	**0.01**

Note: Model fitness based on the likelihood ratio (all models with p < 0.0001)

^1) ^Model outcome: health facility immediate use (HF use) (same day or next day)

^2) ^Model outcome: health facility and antimalarial immediate use (HF AM) (same day or next day)

^3) ^Model outcome: antimalarial immediate use (AM not HF) (same day or next day) not from the health

*illness identified with *degedege *in the illness narratives even though identified primarily with *homa *and malaria

### Treatment-seeking for *homa*, malaria and *degedege*

The demographic characteristics of children in correlation with the outcome variables are presented in Table 3. The sex of the child (female) was associated with less timely health facility use and receipt of anti-malarial medication from the health facility. Being a farmer was also a factor associated with less timely health facility use, but since 95% of the sample were farmers, this could be expected. It was surprising that health facility availability was associated with timely anti-malarial use not from health facility (AM not HF). This is also reflected in the fact that a lack of immediate use of a health facility was observed among children who reported 'timely used anti-malarials not obtained from health facility'. Illness narratives revealed that health facility availability did not motivate caretakers to seek prompt treatment, since they believed that the health facilities would be out of drug stock.

Most of the determinants for 'HF use' and 'HF AM' are similar (Table 3). For the categories of distress, 'no interest to play', 'cough' and 'difficult breathing' were associated with timely health facility use and receipt of anti-malarial from the facility (HF AM), while 'hot abdomen' related more to timely health facility use (HF use). For the perceived causes, people stated that 'eating leftover food' and 'stage of child growth' were more associated with 'HF AM' while 'hereditary causes' and 'bird *degedege*' were associated with less use of 'HF AM'. On the other hand 'blood weakness' was related to 'timely health facility use and receipt of anti-malarial' (HF AM), while those who mentioned 'contamination' as the cause were less likely to use 'timely health facility and receive anti-malarial' (HF AM).

For the outcome variable 'AM not HF', the categories of distress associated with this response included: 'not happy', 'diarrhoea' and 'shivering'. Whereas, the categories of distress of: 'sleeps', 'hot abdomen' and 'froth in mouth' were the symptoms related to less use of timely anti-malarial not from the health facility (AM not HF). The perceived causes related to the same outcome variable were comprised of: breast-feeding and 'do not know the cause', while bird '*degedege*' was associated with less use of 'AM not HF'.

### Treatment-seeking for *degedege*

More specifically, the study narrowed its focus on treatment-seeking patterns for children with *degedege *(Table 4) compared to *homa*. For a general fever case definition, the same children had been studied within the ACCESS Programme by Hetzel *et al *[30], whose interest was to capture the general obstacles to prompt malaria treatment. The present study focuses on how illness experiences and meanings given to *degedege *influence treatment-seeking patterns.

**Table 4:** Demographic and socio-cultural features of children with *degedege *(n = 135)

	HF Use ^1)^ 71.1%		HF AM ^2)^ 66.7%		AM not HF ^3)^ 10.4%	
	Estimate	P-Value	Estimate	P-Value	Estimate	P-Value
**Demographics**						
Malaria*	1.14	**0.04**	1.18	**0.03**		
Homa**			1.44	**0.03**		
HF availability					1.24	0.10
Age			0.43	0.06		
**Categories of distress**						
No interest to play	1.16	0.12	2.52	**0.00**		
Not happy	1.55	0.10				
Sleeps	-1.37	**0.02**	-1.05	0.06		
No strength	0.52	0.15				
Hot abdomen	0.79	**0.02**	0.56	0.05		
Periodic fevers					-2.63	**0.03**
Cough	1.50	**0.01**			1.56	0.06
Difficult breathing	0.87	**0.04**	1.55	**0.00**		
Diarrhoea			0.55	0.14	1.50	**0.02**
Vomiting	-0.55	**0.05**			0.72	**0.04**
Shivering					0.78	0.09
Twitching					1.44	**0.00**
Body becomes stiff			0.42	0.09		
Easily startled					-5.97	**0.00**
**Perceived causes**						
Impure water			0.82	0.13		
Starchy food	-2.41	0.05				
Stage of child growth	1.05	0.09	0.81	0.07		
Constitution/blood weakness	3.03	**0.01**	2.95	**0.00**		
Sanitation/dirty environment	1.54	**0.00**				
Personal hygiene/not keeping clean					4.19	**0.01**
Cold weather					1.39	**0.05**
Spirits	-1.72	**0.03**				
Failure to abstain from sex (parent)	-2.84	**0.03**	-1.13	0.17		
Bird/insect called *degedege*			-0.60	**0.00**		
Other mention					-0.55	0.08

Note: Model fitness based on the likelihood ratio (all models with p < 0.0001)

^1) ^Model outcome: health facility immediate use (HF use) (same day or next day)

^2) ^Model outcome: health facility and antimalarial immediate use (HF AM) (same day or next day)

^3) ^Model outcome: antimalarial immediate use (AM not HF) (same day or next day) not from the health facility

*Illness identified with malaria in illness narratives even though identified primarily with *degedege*

** Illness identified with *homa *in illness narratives even though identified primarily with *degedege*

### 'Timely health facility use" and "timely health facility and anti-malarial use'

The study found some similarities in determinants of the outcome variables 'HF use' and 'HF AM' among children with *degedege*. About 71.1% of all degedege cases were 'timely brought to health facility' and the majority (66.7%) received anti-malarial medication from the facility. In comparison, 45.6% of children with *homa *used timely health facility and 42.7% of them received anti-malarial from the facility. Respondents in these groups also mentioned malaria (p = 0.04) and *homa *(p = 0.03) when talking about the symptoms, indicating that caretakers made a link between convulsions and *homa *or malaria (Table 4).

Symptoms associated with 'HF AM' included: 'hot abdomen', 'cough', 'no interest to play' and 'difficulty breathing'. The following narratives indicate how caretakers explain such symptoms and the need to seek prompt treatment: "*The child's fever was very high, you know always the fever starts in the abdomen then goes to the head then comes degedege, the only thing is to rush the child to health facility*" (female respondent aged 38 from Minepa village). Another caretaker explained, "*the child was coughing and struggling hard to breathe like somebody who has asthma, you know the life of any human being depends on breathing so I was scared that the child might die and took him straight to health facility*" (female respondent aged 24 from Mngeta village). The patterns of distress that were associated with 'less health facility use' were 'vomiting' and 'increased sleeping'. These symptoms were viewed by caretakers as unserious and manageable in the household, by purchasing drugs from drug stores.

The perceived causes associated with 'HF use' and 'HF AM' were 'blood weakness' and 'sanitation/dirty environment'. The following narratives explain the perceived causes of *degedege *by respondents: "*Dirty environment around the house like bushes, grasses, pit, and stagnant water are breeding grounds for mosquitoes causing malaria*" (male respondent aged 35 from Kichangani village). Some caretakers believe that some children are born with weak blood, and that makes them more vulnerable to various diseases, including malaria. As a respondent had put it: "*My child's blood is weak that is why he is prone to many diseases, the only thing I can do is to try and protect her from contacting different diseases otherwise there is nothing I can do*" (female respondent aged 40 from Lupiro village).

'Spirits'(mostly referred by respondents as *shetani *in Kiswahili or witches) and the bird/insect called '*degedege*' were the perceived causes of *degedege *associated with 'less timely use of health facility and receipt of anti-malarial' among *degedege *children. This reveals that 'traditional beliefs' about the cause of *degedege *do influence timely treatment-seeking of health facility. However, illness narratives also revealed that caretakers did not see the importance of prompt treatment at health facilities, since they believed that medical facilities were frequently out of medications. The following translation is typical of quotes from respondents on the reasons not to take *degedege *children to a health facility: "*When I saw my child twitching, I went to the drug shop to buy some anti-malarials, I could not go to the health facility since the drugs are always out of stock*" (female respondent aged 37 from Iragua village).

Another respondent said: "*You see, it is waste of time going to the dispensary hoping to get some drugs and being told to get them from drug shops; sometimes you are also told to buy some syringes and bring them to the dispensary for injection, so when the child started twitching and becomes stiff, I with my mother rushed the child to the drug shop and got some quinine*" (female respondent aged 30 from Mbingu village).

Illness narratives show that caretakers are well informed about the importance of prompt treatment at a health facility, particularly for young children. The knowledge of this had been acquired from various sources, including: MCH (Mother and Child Health) clinics, previous experience with *degedege *cases, HDSS staff and the extensive social marketing activities in the study area [35]. The following is typical of narratives identifying the sources through which caretakers acquire knowledge about malaria: "*I myself know that whenever the child has various symptoms, I have to take her to the dispensary because when we attend the MCH clinic, we are told how to identify malaria symptoms through which we can promptly seek treatment at the dispensary*" (female aged 28 from Idete village).

"*My child had high fever, his stool was red and the eyes used to go up – you know I lost one of my children with the same symptoms, so this time I decided on my own to take the child to the dispensary because the other child passed away while my grandfather was trying to sponge him with traditional medicines. I did not want something bad to happen to my child, I immediately rushed him to the dispensary, he got some treatment and he was healed*" (female respondent aged 34 from Iragua village).

"*People from STIFL (Swiss Tropical Institute Field Laboratory, currently known as Ifakara Health Institute) are doing a wonderful job, you see every house has a number, they visit us frequently and educate us about health issues including malaria and the importance of seeking treatment at a health facility*" (male respondent aged 46 from Mbingu village).

### 'Timely anti-malarial, not from the health facility'

About 10.4% of children who had *degedege *used 'AM not HF' compared to 38.8% of those who had *homa*. The patterns of distress associated with this category include 'diarrhoea', 'twitching' and 'vomiting'. Typical *degedege *symptoms were grouped together and included 'twitching', 'stiff body', 'delirium', 'eyes turn white', 'kicking of legs and arms', 'froth in mouth', 'mouth twisted sideways', 'falling down' and 'easily startled'. Yet, 'twitching' was the only typical *degedege *symptom associated with timely anti-malarial, not from the health facility (Table 4). On the other hand, 'easily startled' was the pattern of distress that has a negative correlation with "timely anti-malarial receipt but not from the health facility" (AM not HF). The perceived causes that were associated with 'timely anti-malarial use not from the health facility' (AM not HF) were 'personal hygiene' and 'cold weather'.

Representative narratives revealed that the major reasons for children with *degedege *receiving timely anti-malarial without seeking care from health facility include a belief in 'lack of drugs' and 'poor diagnosis at health facility'. 'Lack of money to pay for treatment' was also pointed out as a reason not to attend a health facility. The following narratives indicate how caretakers explained reasons for not attending health facility: "*I went straight to buy drugs at drug shop, I can not go to the dispensary since there is no diagnosis there, they do not take your blood to see what you are suffering from, so even the doctor does not know what he is treating*" (male respondent aged 41 from Mpofu village). A woman explained: "*There is no need to go the dispensary because in the end you will be told to purchase drugs from drug shops since the dispensary is always out of stock*" (female respondent aged 21 from Lupiro village). Another caretakers explained: "*I did not have money, I have no work that gives me any income, how can I go to the dispensary with only 100 shillings?, I just went to drug shops, still the money was not enough for the drugs *" (female respondent aged 18 from Ikule village).

Although some caretakers also used traditional treatment as the first aid for *degedege*, they recognized the importance of timely modern treatment to cure *degedege*. The following quote illustrates the experience reported: "*When I saw my child twitching, I went to the bush and got some leaves called 'manunganunga', which I used to sponge the child, after few minutes the twitching stopped so I took my child to the drug shop and we got an anti-malarial*" (female respondent aged 39 from Mpofu village).

## Discussion

The study examined local understandings and treatment practices of *degedege *compared to fever for children younger than five years of age, from the perspectives of their caretakers. This study applied an integrated methodology that examined health system, livelihood, as well as cultural epidemiological determinants of behaviour. The strength of this study lies in the fact that it does not only describe "conventional" factors influencing treatment-seeking behaviour for malaria (e.g. socio-economic characteristics), but also elucidates the significance of illness experiences, meanings and resulting treatment-seeking behaviour, in combination with perceptions on health service delivery. A combination of factors explains treatment-seeking behaviour and is hence key for targeted disease control.

Findings from this study provide both parallel and diverse ideas to previous studies about the role of illness experience, and its meaning for treatment practices of *degedege *and fever cases. The link between *degedege *and malaria, that respondents make, is certainly confirmed in this study, unlike other studies in sub-Saharan Africa, as reviewed by William and Jones [6], and in Tanzania in particular, where the two conditions were perceived as distinct [14-18].

The majority of children with *degedege *were taken to health facilities in a timely manner (71.1%), compared to those who had *homa *or malaria (45.6%). The finding confirms the study by de Savigny *et al *[21] which indicates modern care as the first choice of treatment for 78% of fatal malaria cases, and that by Ahorlu [22] where 90% of respondents identified convulsions as severe condition and recognized the need to seek modern treatment. This finding supports that at least in the selected research area there has been a shift in the practices for first treatment of *degedege*, compared to what has been reported in previous studies for the same area [14-19]. Moreover, the results provide information on the differences between the treatment patterns for fever (*homa*) and *degedege*. Fever cases have been reported as resorting more to modern treatment approaches than *degedege *cases [15,18], while the present study found a higher percentage children with *degedege *(71.1%) than children with fever (45.6%) accessing modern treatment. A number of studies have shown that treatment delay for fever/malaria is due to the perception that this illness is a 'normal everyday illness', and thus not a serious one [7,24,36].

While *degedege *symptoms were regarded as belonging not to the modern treatment field [14,18], this study attests the use of modern treatment for *degedege *cases. However, these results can also be attributed to the fact that people in the study area have been exposed to multiple health education campaigns since 1970, including the national '*Mtu ni Afya' *(meaning "Man is Health") campaign, which provided intensive information about symptoms, causes, treatment and prevention of malaria [13]. Moreover, in 1996, the study area was marketed via the thorough insecticide-treated nets social marketing campaign [17]. That campaign was preceded by a study [17], which revealed that communities did not link malaria with *degedege*, and as a result these findings were incorporated in the social marketing to correct the knowledge on linkage between the two. On the other hand, accurate knowledge does not essentially translate into appropriate behaviour, since treatment-seeking is also influenced by a number of other factors [37].

Caretakers of children with *degedege *who did not use health facilities at all, were less driven by local ideas with regard to the importance of traditional treatment as indicated by various studies [7-12,14-18], but instead, by the fact that the health facilities do not always provide adequate treatment options, including drugs and proper diagnosis, or that communities lacked the money to pay for treatment, confirming results of a study by Thera [24]. That health facilities are more often used may result from traditional ideas having become less pervasive, or from the fact that in the past there were fewer, or even no health facility options. Yet, the number of health facilities in Tanzania has been comparably high since 1991, when private health facilities were allowed to operate on their own. It is also possible that in areas where a health facility had previously been available, but not used, the association of traditional causes, however valid, was not as influential on behaviour even then.

A number of studies reported delayed health facility attendance and anti-malarial injection for various *degedege *related symptoms, including eye rolling, body stiffening and foaming at the mouth, which was regarded as fatal and therefore required traditional treatment [14,18]. On the contrary, these findings demonstrate that illness experience of *degedege *did not hinder caretakers to *timely *approach health facilities and receive anti-malarial medications. Instead, rather symptoms related to *homa*, including vomiting and increased sleeping, correlated more with less timely use of health facility than *degedege*. This particular finding corresponds with results presented by Muela [13], who found that for uncomplicated malaria, vomiting was perceived as a sign of relief, rather than a symptom showing an ascending to complicated malaria, and therefore treatment delay was common.

Spirits, the insect '*degedege*', and non-abstinence from sex were the perceived causes of *degedege*, and associated with less timely use of health facility and receipt of anti-malarial. This finding confirms views from previous studies, where *degedege *was related by respondents to spiritual causes and hence less likely to trigger the use of health facilities [7-12,14-19]. However, these ideas were not so influential as to keep the majority of children with *degedege *from accessing health facilities and receiving anti-malarial medication. More importantly, the so called dirty environment (bushes, grasses, stagnant water) were considered as contributing to breeding sites for mosquitoes, which were recognized as the cause of *degedege *and accelerated the need for prompt treatment. This confirms a study conducted within the same programme [30], which found that for fever and *degedege *cases, mosquito bites were the most cited perceived cause.

More generally, the findings indicate that symptoms rather than causes are now the drivers of health-seeking behaviour. This may at least partly be attributed to the many health interventions and campaigns carried out in the study area emphasizing the importance of recognizing and responding to danger symptoms. The present study took place two years after the 2002 introduction of IMCI (Integrated Management of Childhood Illnesses) devoting training to caregivers on early recognition of dangerous symptoms among children, through which prompt treatment at health facility was highly suggested [38]. The social marketing of insecticide treated nets (KINET) and of prompt and appropriate treatment seeking (ACCESS) program have certainly also contributed.

However it is important to point out that the results of the study are context-based and cannot be generalized. Firstly, the sample size of the study was restricted since it focused on first treatment and was based only on reported fever and *degedege *cases. Secondly, the study site was influenced by extensive malaria research activities and interventions that have been taking place in Kilombero and Ulanga Districts since several decades. While the results might not reflect the situation in other parts of the country, they provide insights into the effects of interventions such as the ACCESS programme in this area, and may thus inform research, policy and practice.

## Conclusion

This study examined caretakers' treatment-seeking patterns for children with *degedege *and *homa*, and then compared the findings with results from previous studies focusing on the same theme. Findings suggest that intensive community health education on causes and danger symptoms can trigger seeking of appropriate and timely treatment, and had positive influence on changed health-seeking behaviour in the study area. Health facility use was even higher among *degedege *children compared to children with normal fever. Furthermore, findings revealed that traditional ideas are no longer a significant influence in *delaying *health facility use for children with *degedege*. Even for children with *degedege *who did not use a health facility at all, reasons associated with this had more to do with poor services in the respective facility, and/or lack of money to pay for consultation and/or treatment, than the belief that *degedege *is better managed by traditional practices.

The main reasons why services were considered as inadequate were a lack of drugs and competence as well as equipment to make accurate diagnosis. Communities are exposed to insecurities when they lack financial capital, a significant livelihood asset in seeking care, especially when the exemption of children under the age of five who are supposed to receive free treatment in health facilities is not effective [30]. In order to further encourage people to seek timely and appropriate treatment, quality of care at health facilities needs to be improved, through making diagnosis and provider compliance with treatment guidelines more accurate, as well as therapies including drugs more available and affordable to communities. Initiatives for strengthening livelihoods including economic capabilities are also relevant in ensuring prompt and timely malaria treatment. Moreover, further education needs to focus on proper causes of malaria, specifically to correct the inaccurate association of malaria with *degedege *bird and hereditary causes. Finally, the correlation between female children and less use of timely health facility certainly needs to be explored in more depth since this may reflect a gender bias with regard to early treatment.

## Competing interests

The authors declare that they have no competing interests.

## Authors' contributions

AD was involved in the design and implementation of the study, field work, data management, analysis, interpretation and writing of the manuscript. AS, BO, CL, HM conceived the programme and its components, provided technical support and supervision, and commented on the manuscript. MW was involved in the design and analysis of the EMIC, interpretation and discussion of the findings. BO was involved in the conception and design of the study, data analysis, interpretation and writing of this paper. CP was involved in data analysis, interpretation and writing of this paper. MH was involved in the design and implementation of the studies and IM contributed to data collection and discussion of the findings. DG was in charge of statistical support and contributed to the analysis of the data. FK, AM and CM were responsible for the development and implementation of the interventions. All authors have read and approved the final manuscript.

## Ethical review

This paper was published with the permission of Dr. Andrew Kitua, Director General, National Institute for Medical Research. Ethical Clearance of the ACCESS Programme proposal was granted by the National Institute for Medical Research of the United Republic of Tanzania (NIMR/HQ/R.8a/Vol.IX/236, September 16, 2003).
